# Insights into Early Pregnancy Demise following Intracytoplasmic Sperm Injection in Women with Unexplained Infertility

**DOI:** 10.5935/1518-0557.20220005

**Published:** 2023

**Authors:** A Bahaa Eldin, M Ibrahim, A Elsheikh, A Awad, A Elsadek, H Fekry, N Ali

**Affiliations:** 1 Department of Obstetrics and Gynecology, Ain Shams University, Cairo, Egypt; 2 Department of Obstetrics and Gynecology, Al-Azhar University, Cairo, Egypt

**Keywords:** age, embryo transfer, intracytoplasmic sperm injection (ICSI), obesity, early pregnancy loss

## Abstract

**Objective:**

To investigate the effects of some potential risk factors on early pregnancy loss -EPL - in a cohort of pregnant women treated by assisted reproductive technology - ART.

**Methods:**

This is a retrospective cohort of 195 pregnancies, defined as serum human chorionic gonadotrophins ≥ 10 IU/l on day 14 - 17 after embryo transfer, recruited from an assisted reproductive technology unit, Ain Shams & Al-Azhar Maternity hospital, Cairo, Egypt, during the period from January 1^st^, 2016 to December 31, 2020. Risk factors investigated were maternal age, body mass index, status, baseline hormonal profiles, treatment protocol, quality and number of embryos.

**Results:**

Overall early pregnancy loss among the studied 195 pregnant women was 29 cases (15%). The risk of early pregnancy loss was associated with older age and fewer number of embryos transferred. Women > 35 years were found to have two and half times of early pregnancy loss compared with younger age group < 25 years, this was not significant after adjusting for other factors. The risk in both lean (BMI < 25 kg/m^2^) and very obese (BMI > 35 kg/m^2^) women was also not significantly higher in unadjusted analysis. Transfer of two or more embryos was associated with a non-significant reduced risk of early pregnancy loss, and after adjusting for other factors, the reduction was about 70%.

**Conclusions:**

Early pregnancy loss represents a considerable drawback of intracytoplasmic sperm injection treated women with old age to increase the risk of early pregnancy loss and transferring more than one embryo to reduce the risk by about 70%. Obesity and other factors appeared to play a minor role.

## INTRODUCTION

A rise in hCG concentration in either urine or serum has been used to detect the establishment of pregnancy within the first few weeks of conception. Failure to confirm the presence of an embryonic sac(s) or fetal heart by subsequent ultrasound scan at 6-8 weeks gestation occurs in 18-22% of all pregnancies (Alfredsson, 1988; Wilcox *et al*., 1988; Kolstad *et al*., 1999) and it is sometimes defined as early pregnancy loss (EPL). In assisted reproductive technology (ART), pregnancy loss before clinical detection by ultrasound scan is also commonly referred to as biochemical pregnancy. While studies carried out in the general population can provide some estimates of the risk of EPL, the sample size of these studies has usually limited the statistical power for a detailed investigation of the potential risk factors for EPL (Wilcox *et al*., 1990). Women treated by ART are routinely monitored for early detection of pregnancy by measuring serum hCG concentrations on a specific day, usually 14-17 days following oocyte retrieval, equivalent to ovulation in the general population, and again by ultrasound scan at about 6-8 weeks gestation. Therefore, They represent an ideal population to study the potential risk factors for EPL, with the results possibly applicable to the general population.

High rates of EPL, ranging from 12 to 48%, have been reported in ART (Barlow *et al*., 1988; Acosta *et al*., 1990; Levy *et al*., 1991; Schmidt *et al*., 1994; Simón *et al*., 1999; Fedorcsák *et al*., 2000; Sugantha *et al*., 2000). EPL significantly reduces the initial success of ART treatment, decreases the efficiency of treatment and increases the psychological burden on patients. However, risk factors for EPL in pregnancies after ART have not been comprehensively studied, despite its apparent significance. With a view to altering practice, an important conceptual issue relates to the mutability of risk factors for EPL, as potentially modifiable factors can form the basis for clinical or preventive interventions. For example, maternal age is not reversible; the age at which women have children is, however, amenable to modification within the population, as indicated by secular trends of increasing maternal age at the birth of the first child. A recent report has linked obesity with greater risk of EPL (Fedorcsák *et al*., 2000); and obesity has been shown to be a detrimental factor for pregnancy rate (Wang *et al*., 2000) and spontaneous abortion (Hamilton-Fairley *et al*., 1992) in ART programs. Obesity is also potentially modifiable, possibly amenable to low cost, non-invasive, self-management by patients. The objective of this retrospective study is to investigate the effect of some potential risk factors for EPL in a cohort of pregnant women treated by ART, International Islamic Center for Population Studies and Research (ICCPSR), Ain Shams Maternity Hospital.

## MATERIAL AND METHODS

During the period from January 1^st^ 2016 to December 31, 2020 a total of 799 cycles were carried out. In these cycles, 195 women had > 10IU/L hCG measured on day 14-17 after embryo transfer. The main stimulation protocols used were long (GnRHa commenced at least two weeks before starting stimulation, and continued up until HCG was administered), short (GnRHa commenced at the same time as starting stimulation and continued up until the day of hCG administration), soft from cycle day 2 or 3 (treatment day 1) 100 mg clomiphene citrate (CC) orally was administered once daily for 7 days. On treatment, day 6 ovarian stimulation was initiated with three ampoules of human menopausal gonadotrophins (HMG) till dominant follicle reached 18 mm) and low cost (100 mg of CC daily orally) from cycle day 3 to 7. A daily injection of hMG 150 iu i.m. was given on the 9^th^ cycle-day, till the dominant follicle reached 18 mm) protocols. After initial confirmation of pregnancy following hCG rise, EPL was ascertained by either a self-reported miscarriage before 6-8 weeks gestation or by an absence of embryonic sac or blank embryonic sac(s) as detected by ultrasound around 6-12 weeks gestation. Pregnancy loss after that was not considered in this study. The risk factors for EPL investigated in this study were maternal age, body mass index (BMI, kg/m^2^), baseline hormonal profiles, protocol types and number and quality of embryo transferred (according to Gardner *et al*., 2000) for clarity of data analysis, the studied variables were categorized during the study analysis. Their ages were classified into four categories as follows: <25 years, 25-30 years, 30-<35 years and >35 years; BMI into three: <25 kg/m^2^ (normal), 25-<30kg/m^2^ (overweight) and >30 kg/m^2^ (obese) (WHO, 2021). The protocol was categorized as long, short, soft and low cost.

The ethical committee of the Ain Shams University Maternity Hospital discussed and approved the study.

### Sample Size Justification

The required sample size was calculated using the G*Power Software (Universität Düsseldorf, Germany). The primary outcome measure was ongoing pregnancy, i.e., the patients’ likelihood that their pregnancies continued beyond 12 weeks. The data was then analyzed using the statistical analysis software package (SAS Institute Inc. Proprietary software release 9.0. Cary, NC, SAS Institute Inc. 2002). We used the Chi square test or the Fisher’s exact test and the t-test as appropriate. We used a multivariate logistic regression analysis to assess all potential risk factors simultaneously. The results were shown as odds ratios (OR) and its 95% confidential interval (CI) was calculated from the logistic regression model. To undergo this model, pregnancy patients with EPL were considered as the cases of this study and those pregnant without EPL were considered the controls.

### RESULTS

Out of the 195 pregnant women, 29 reported early pregnancy loss (EPL). Table 1 presented the characteristics of the studied EPL (=29) and non-EPL (n=166) groups. The mean age of the study cohort at the time of treatment was 30.6±5.7 years in the EPL group and 29.3±5.3 years with no statistically significant difference. The mean BMI was 25.8 and 26.6 kg/m^2^ in the EPL and other group respectively with no statistically significant difference. For other characteristics there also were statistically significant differences. However, the highest percent of EPL occurred with the short protocol and the lowest with the soft and low-cost protocol.

**Table 1:** Characteristics of the studied pregnant women by EPL.

Characteristics	EPL (n=28)		No EPL (n=169)		*p*-value
Age in years (mean ± SD)	30.6±5.7		29.3±5.3		0.20
BMI kg/m^2^ (mean ± SD)	25.8±5.8		26.6±5.5		0.45
FSH (mean ± SD)	6.5±2.1		6.1±2.5		0.30
LH (mean ± SD)	5.1±1.7		5.6±2.8		0.33
PRL (mean ± SD)	14.6±7.3		13.0±8.4		0.30
E2 (mean ± SD)	58.6±43.3		55.4±43.5		0.73
Number of embryo transfer (mean ± SD)	2.6±0.7		2.8±0.5		0.60
Prescribed protocol Long Short Soft Low cost	20 7 0 2	14.0 25.0 0.0 6.0	115 20 2 29	86.0 75.0 100.0 94.0	0.19
Embryo grade Grade A Grade B Mixed A & B	27 0 2	15.0 0.0 13.0	152 1 13	85.0 100.0 87.0	0.90

* Data presented by mean ± SD or by number and %.

Table 2 presented the unadjusted logistic regression model for the association of EPL with the studied factors. For age of the studied women, the findings showed that increasing age would increase the likelihood of EPL with a risk of about twofold among women > 35 years compared to women <25 years (OR=2.00), although not significant. The same result was also observed among women who received the short protocol. For other factors, no effects were observed to greatly affect the probability of EPL, except the number of ET cycle, where increasing the number of ET has been found to decrease the probability of EPL, although not significant. Also, baseline hormonal profiles were found to have no effects (data not shown).

**Table 2:** Unadjusted odds ratio (OR) and 95% confidence intervals (CI) for the association EPL with the studied factors.

Factors	EPL (n=29)	No EPL (n=166)	OR*	95% CI **
Age (years) <25 25-30 30-35 > 35	4 7 10 8	30 59 48 29	1.00 0.90 1.60 2.00	Reference 0.25-3.28 0.45-5.43 0.60-7.60
Body mass index (kg/m^2^) >25 25-30 > 30	4 13 12	28 68 70	1.00 1.30 1.20	Reference 0.40-4.46 0.35-4.01
Prescribed protocol Long Short Soft Low cost	20 7 0 2	115 20 2 29	1.00 2.00 - 0.40	Reference 0.75-5.37 - 0.10-1.80
Embryo transfer Single Two embryos Three or more	3 4 22	7 23 136	1.00 0.40 0.40	Reference 0.10-2.57 0.10-1.60
Embryo grade Grade A Grade B Mixed A & B	27 0 7	152 1 13	1.15 - 1.00	0.25-5.40 - Reference

* OR=odds ratio.

** CI=confidence interval

Table 3 presented the predictor multivariate logistic regression model for the association of EPL with the studied factors. According to the results of the predictor model, age and ET number are the only two variables found to predict the occurrence of EPL. In the multivariate model with controlling for the effect of other factors, the probability of EPL is increased by two and half times among older women with age > 35 years, although not statistically significant. The adjusted OR for these women was 2.50 and 95% CI was 0.55-8.40. The probability, however, is reduced by about 70% among women transferred by more than a single embryo (OR=0.3) in women transferred by two or three and more embryos, although not statistically significant.

**Table 3:** Adjusted odds ratio (OR) and 59% confidence intervals (CI) for the association EPL (predictor multivariate logistic model).

Factors	EPL (n=29)	No EPL (n=166)	OR*	95% CI **
Age (years) <25 25-30 30-35 > 35	4 7 10 8	30 59 48 29	1.00 0.95 1.70 2.50	Reference 0.20-4.12 0.40-6.13 0.55-8.40
Embryo transfer Single Two embryos Three or more	3 4 22	7 23 136	1.00 0.30 0.30	Reference 0.10-3.42 0.10-2.53

* OR=odds ratio; OR adjusted by BMI, protocol, embryo grade and basal hormones

** CI=confidence interval

## DISCUSSION

Of the 195 studied pregnancies following ICSI cycle treatment, there was a 15% EPL in this population cohort. There is a high wastage of pregnancy as EPL in both the general population and in the ART population. In this study, the findings revealed that older age in women increases the risk of EPL by about two and half times, while transferring more than two embryos reduce this risk by about 70%. The risk of EPL, 15% reported in this study, is lower than the estimates given for the general population. However, direct comparison of the two populations can be problematic, since more sensitive assays using earlier hCG measurement and lower hCG criterion for pregnancy detection have been utilized in the general population studies (Wilcox *et al*., 1988). Another possible factor that may complicate the early detection of pregnancy in ART is the common use of large doses of hCG for inducing ovulation or luteal support. Nonetheless, an earlier study suggested that residual hCG from either ovulation induction or luteal support would have been reduced to an undetectable level at the time of early pregnancy detection (Lenton *et al*., 1988). It is likely that the real risk of EPL would be higher in the ART population than in the general population, although direct evidence to support this assertion is yet to be obtained. In fact, many methodological differences also exist between the reports of EPL in the ART population, which may account for the varying risks of EPL reported. The risk of EPL of 15% reported in this study, by and large, is within the range of a few other reports of ART population using similar criteria (Simón *et al*., 1999; Fedorcsák *et al*., 2000). The level of hCG used here for pregnancy detection was well within the hCG assay sensitivity, although it was lower than the common clinical criterion for positive pregnancy (>30-50IU/L), which is usually for informing patients and is considered conservative. On the other hand, since the criterion used in this study, hCG > 10 IU/L at day 14-17, was much higher than the used in the general population studies (Wilcox *et al*., 1988), it can be anticipated that some very early pregnancy loss would not have been counted.

Couples who are planning their pregnancies want to know the potential hazards, while those receiving infertility treatment will try to avoid any potential risk factors causing pregnancy loss. Since EPL causes a big reduction in the initial ART success, it reduces its efficiency and increases the burden of psychological stress to infertile couples. Therefore, establishing the risk factors for EPL has important clinical implications in improving the efficiency of ART treatment, in addition to increasing the understanding of possible mechanisms of early pregnancy failure.

The lack of effect of young maternal age on EPL appeared to be consistent with that reported in two previous studies (Wilcox *et al*., 1990; Dickey *et al*., 1993). Alternatively, and consistent with this study results, Balmaceda *et al*. (1994) suggested that there is an increased incidence of EPL and chromosomal abnormalities in oocytes from older women. In this study, women aged 35 years, and more were found to have a higher risk of EPL compared with women <25 years. It is possible that embryos in older women may have lost their ability to develop even before implantation, although the uterus may have the capacity to conceive (Sauer *et al*., 1992; Balmaceda *et al*., 1994; Sauer, 1997).

In this study, the results showed that transferring more than one embryo is associated with decreased risk of EPL. The reduction of risk was about 70%. However, the practice of multiple embryo transfer in ART also means that some EPL may be hidden by the continuing growth of companion embryo(s) (Simón *et al*., 1999; Fedorcsák *et al*., 2000). However, the results of our study showed a minimal effect of the other studied risk factors on EPL in our cohort. For BMI, the risk is slightly increased in overweight women with a BMI between 25 and 30 kg/m^2^ and obese women with BMI > 30 kg/m^2^. This appears to be consistent with results reported recently (Fedorcsák *et al*., 2000). The association between embryo quality and the risk of EPL is another interesting finding which needs to be confirmed, where no effect was found regarding the A and B quality of the transferred embryos. In contrast to such finding, it is known that the selection from large number of embryos available leads to a better clinical pregnancy rate. The lack of relationship between the quality of ET and the risk of EPL was supported by one earlier study (Dickey *et al*., 1993).

There may be other possible risk factors that should be considered, such as smoking (Dickey *et al*., 1993) or bacterial vaginosis, which is associated with adverse pregnancy outcomes across all gestational ages (Ugwumadu, 2002). Lack of data, due to the retrospective nature of the study, prevented us from adjusting for their possible effect and this may have reduced the sensitivity of the study. Also, lack of data about psychological stress prevents us to investigate its effects on EPL. Missing data regarding the different causes of infertility (>50%) also hamper the examination of this important factor effect.

Although we couldn’t investigate the mechanism of EPL in this retrospective study, it has been suggested that EPL may be due to failure of the maternal support system or fetal impairment. Due to the extreme difficulty in obtaining pregnancy material in EPL, the determination of either pathological or a chromosomal cause is hard. A recent review (Norwitz *et al*., 2001) has discussed in detail early implantation, implantation failure and the possible mechanism involved.

## CONCLUSION

In summary, this study has investigated some factors and their potential association with the risk of EPL and found that older age of women is associated with an increased risk of EPL, while the increased number of ET decrease the risk. Obesity, ET quality and other factors affecting the risk of EPL was not evident. Further studies are needed to confirm or refute the findings of this study.
